# Genetic Diversity, Rather than Cultivar Type, Determines Relative Grain Cd Accumulation in Hybrid Rice

**DOI:** 10.3389/fpls.2016.01407

**Published:** 2016-09-21

**Authors:** Liang Sun, Xiaxu Xu, Youru Jiang, Qihong Zhu, Fei Yang, Jieqiang Zhou, Yuanzhu Yang, Zhiyuan Huang, Aihong Li, Lianghui Chen, Wenbang Tang, Guoyu Zhang, Jiurong Wang, Guoying Xiao, Daoyou Huang, Caiyan Chen

**Affiliations:** ^1^Key Laboratory of Agro-Ecological Processes in Subtropical Region, Institute of Subtropical Agriculture, Chinese Academy of SciencesChangsha, China; ^2^Institute of Rice Science, Hunan Agricultural UniversityChangsha, China; ^3^Yuanlongping High-Tech Agriculture Co., LTDChangsha, China; ^4^China National Hybrid Rice R&D CenterChangsha, China; ^5^Lixiahe Agricultural Research Institute of Jiangsu ProvinceYangzhou, China; ^6^Beishan Agricultural Service Center of Changsha CountyChangsha, China

**Keywords:** cadmium, hybrid rice, *indica*, QTL, genetic diversity

## Abstract

Cadmium (Cd) is a toxic element, and rice is known to be a leading source of dietary Cd for people who consume rice as their main caloric resource. Hybrid rice has dominated rice production in southern China and has been adopted worldwide. The characteristics of high yield heterosis of rice hybrids makes the public think intuitively that the hybrid rice accumulates more Cd in grain than do inbred cultivars. A detailed understanding of the genetic basis of grain Cd accumulation in hybrids and developing Cd-safe rice are one of the top priorities for hybrid rice breeders at present. In this study, we investigated genetic diversity and grain Cd levels in 617 elite rice hybrids collected from the middle and lower Yangtze River Valley in China and 68 inbred cultivars from around the world. We found that there are large variations in grain Cd accumulation in both the hybrids and their inbred counterparts. However, we found grain Cd levels in the rice hybrids to be similar to the levels in *indica* rice inbreds, suggesting that the hybrids do not accumulate more Cd than do the inbred rice cultivars. Further analysis revealed that the high heritability of Cd accumulation in the grain and the single *indica* population structure increases the risk of Cd over-accumulation in hybrid rice. The genetic effects of Cd-related QTLs, which have been identified in related Cd-QTL mapping studies, were also determined in the hybrid rice population. Four QTLs were identified as being associated with the variation in grain Cd levels; three of these loci exhibited obvious *indica*-*japonica* differentiations. Our study will provide a better understanding of grain Cd accumulations in hybrid rice, and pave the way toward effective breeding for high-yielding, low grain-Cd hybrids in the future.

## Introduction

Cadmium (Cd) is a highly toxic heavy metal that has accumulated in agricultural paddy soils and threatens human health via the soil-to-crop pathway (Chaney, 1980; Satarug et al., 2010). As an important staple crop feeding nearly half of the world's population, rice tends to accumulate high levels of Cd in the grain without yield losses (Chaney et al., 2004). As a result, Cd contaminated rice and its derivatives are a leading source of Cd for people who depend on rice as a basic food staple (Dudka and Miller, 1999; Clemens and Ma, 2016). To reduce the effects of Cd on human health, the Codex Alimentarius Commission of the FAO/WHO has established a maximum Cd limit of 0.4 mg/kg in polished rice for human consumption. In China, the maximum allowable level of Cd in rice grain is 0.2 mg/kg. Although progress has been made in pollution control and phytoremediation to reduce the Cd content in soil, problems were inevitable because of the high costs involved and the occupation of farmlands (Raskin et al., 1997; Yu et al., 2006; Zhang, 2015). To mitigate Cd contamination in rice grain without compromising rice production, Chinese scientists have developed an integrated control measure known as “VIP” to reduce the Cd levels in grain; low Cd varieties (V), reasonable irrigation (I), and increasing the field pH (P) (Wang et al., 2016). Of these three components, development of low Cd varieties is preferred because it is the most cost effective.

Hybrid rice has dominated rice production in southern China and has been adopted worldwide, offering up to a 30% yield advantage over the best conventional varieties due to its outstanding heterosis (Yu et al., 1997; You et al., 2006; Zhou et al., 2012). Hybrid rice cultivation dominates in the Yangtze River Valley of China and has expanded to more than 30 countries worldwide, and more than half of the rice-growing land in China is devoted to cultivation of hybrid rice (Li et al., 2005; Lin et al., 2015; Singh et al., 2015). In hybrid rice breeding programs, breeders have made great advancements toward improving agronomical traits, especially those related to yield, and a number of elite commercial combinations have been obtained (Yuan and Wu, 2004; Li et al., 2007). However, levels of biologically-available Cd in the subtropical region, where high-yield hybrid rice is mainly planted, are relatively high in the soil; consequently, there is an increased risk of high levels of Cd accumulating in the grain (Zhao et al., 2014; Wang et al., 2016). Comparisons of grain Cd levels between high-yielding hybrids and traditional cultivars in previous reports have reinforced the need to reduce grain Cd in the hybrids (Gong et al., 2006; Chen et al., 2008). At present, genetic improvements in hybrid rice specifically directed at reducing grain Cd accumulation have become a top priority for hybrid rice breeders.

Genotypic variations in Cd accumulation and its underlying genetic mechanism have been a focus for both the breeder and geneticist to reduce Cd accumulation in rice grain (Clemens and Ma, 2016). The translocation, compartmentalization, and grain accumulation of Cd in rice varies greatly not only between the subspecies of *Oryza sativa*, but also among cultivars within a single subspecies (Morishita et al., 1987; Ishikawa et al., 2005; Liu et al., 2005; Zeng et al., 2008; Yan et al., 2010). It is generally acknowledged that *Japonica* rice varieties accumulate less Cd in the grain than do *Indica* rice varieties. However, no consistent results have been obtained from the comparisons of Cd accumulation in hybrid rice with that in traditional inbred cultivars. Some reports support the assumption that hybrid rice with high yield heterosis accumulates more Cd than do the inbred cultivars (Gong and Pan, 2006; Chen et al., 2008), while others have suggested that there is no evident divergence between these two rice types in terms of Cd accumulation (Liu et al., 2005). Genetically, a group of QTLs for cadmium absorption, translocation, and/or accumulation has been identified to explain the observed genotypic variation in rice (Ueno et al., 2009; Ishikawa et al., 2010; Yan et al., 2013; Zhang et al., 2013). One of these, *OsHMA3*, the QTL-gene for qCd7, has been cloned. Natural missense mutations in the functional gene *OsHMA3* led to the hyper-accumulation of grain-Cd in rice (Ueno et al., 2010, 2011; Miyadate et al., 2011; Yan et al., 2016). These studies improved our understanding of the natural variations in Cd accumulation and its genetic dependence. However, unfortunately, no study has focused on commercial hybrid populations, and the effects of these mapped QTLs in hybrid rice are still unknown.

In our previous study, we performed an incomplete diallel mating design to analyze the effect of heterosis on rice grain Cd accumulation, and found that additive parental genetic effects determined the grain-Cd level in the hybrids (Yao et al., 2015). To further elucidate the genotypic variations and the genetic mechanism of grain-Cd accumulation in hybrids in a broad genetic background, we examined the performance of 617 elite hybrid varieties collected from the middle and lower Yangtze River Valley in China and compared it with that of 68 worldwide inbred cultivars in Cd polluted paddy fields. By investigating the population structure and the accumulation of Cd in the grain, we found that most of the hybrids tested have the *Indica* genetic background which has a high heritability of grain-Cd accumulation. We then performed an association analysis with 14 Cd-related QTLs identified from different genetic populations to identify the effective QTL loci that affect grain Cd accumulation in the hybrids. The goal of our study is to provide a better understanding of grain Cd accumulation in hybrid rice, which will facilitate the breeding of Cd-free hybrid rice in the future.

## Materials and methods

### Plant material and field conditions

In this study, 617 *Indica* hybrid varieties were collected from hybrid rice-growing regions in southern China to investigate grain Cd accumulation. The majority of them were elite commercial hybrids that are presently grown. All of the hybrids were planted in two different Cd-polluted paddy fields (Beishan, China) in the 2014 rice-growing season in the experimental fields of the Institute of Subtropical Agriculture of the Chinese Academy of Sciences (ISA) in Changsha, China. The soil Cd concentration was 1.80 mg/kg in field I and 2.45 mg/kg in field II, with pH values of 5.4 and 5.5, respectively. To determine the differences in grain Cd levels between inbred rice cultivars and hybrids, a total of 68 inbred rice (*Oryza sativa*) varieties, obtained from the International Rice Research Institute (IRRI), were used. All varieties can be clustered into two subspecies, such as *Indica* and *Japonica*, and five ecotypes, such as *indica* (*ind*), *aus, aromatic* (*aro*), *temperate japonica* (*tej*), and *tropical japonica* (*trj*) (Garris et al., 2005). The rice cultivars were planted in three different Cd-polluted paddy fields (Zhuzhou and Beishan) from 2012 to 2014. The soil Cd contents of the three fields were 2.80, 1.90, and 1.80 mg/kg, with pH values of 5.2, 5.0, and 5.4, respectively. The rice hybrids and cultivars were planted in the same fields in Beishan in 2014.

The experiments were arranged in a randomized complete block design with three replicates with a spacing of 17 cm between plants and a distance of 20 cm between rows in each field. Field management followed normal agricultural practices except that intermittent irrigation was adopted to maximize the phenotypic differences.

### Determination of Cd and four other metal elements

Rice grain was harvested and air-dried to reduce the water content. After milling the grains into brown rice, samples of 200.0 g were ground into powder and oven-dried at 80⋅C to constant weight for determination of Cd concentrations. For wet digestions, 2.0000 g subsamples were placed in an acid mixture of HNO_3_-HClO_4_ (6:1, v/v). The process of wet digestion was similar to that in our previous study (Yao et al., 2015). Blank digests with no samples and the certified standard material samples (CRM rice; GBW10045, GSB-23) were also treated the same way. The concentrations of Cd and four other metal elements, i.e. Fe, Zn, Mn, and Cu, were determined on an inductive coupled plasma emission spectrometer (Agilent Technologies 700 Series ICP-OES, USA), using the operating parameters as recommended by the manufacturer. Certified standard material (rice flour) was used to ensure the precision of the elemental analysis. Each sample was run in duplicate to ensure the repeatability of the results.

### Population structure and QTL-dependence-associated analysis in the hybrids

To investigate the genetic background of hybrids, a population structure analysis was performed. We used a total of 36 markers, including 22 simple sequence repeat (SSR) and 14 insertion-deletion (In/Del) markers; the marker loci are evenly distributed on the 12 chromosomes and were selected based on the rice genetic map (Temnykh et al., 2001). The sequences of these marker primers were designed based on the Gramene data base (http://archive.gramene.org/; Supplemental Table 3).

The genomic DNA from each hybrid was extracted using a modified potassium acetate-SDS protocol (Dellaporta et al., 1983). PCR amplifications were performed using standard protocols with minor modifications. The PCR products of the SSR markers were separated and sized by capillary electrophoresis using the DNA analyzer (Fragment Analyzer INFINITY™, Advanced Analytical Technologies, Inc., USA). The products of In/Del markers were separated and visualized on 4% polyacrylamide denaturing gels or 4% agarose gels to distinguish allelic polymorphism.

STUCTURE 2.3.4 was used to evaluate the population structure with a maximum likelihood method by a burn-in of 10,000, a run length of 100,000, and a model allowing for admixture and correlated allele frequencies (Pritchard et al., 2000). Three independent runs yielded consistent likelihoods of the population structure for each predicted K value. The neighbor-joining (NJ) tree was constructed based on a distance matrix calculated by PowerMarker 3.25 (Liu and Muse, 2005). MEGA 5.0 (Tamura et al., 2011) was used to generate the phylogenetic tree.

Based on previous reports (Ueno et al., 2009, 2010; Ishikawa et al., 2010; Yan et al., 2013; Zhang et al., 2013), a total of 14 QTLs (24 linkage markers) were utilized and genotyped in hybrid rice (Supplemental Table 4). The Q matrix was obtained with STRUCTURE (version 2.3.4) and the Kinship matrix was obtained with SPAGeDi 1.5 (Hardy and Vekemans, 2002). Based on inferred ancestry of the hybrids (Q matrix) and the relative kinship (K matrix), a general linear model (GLM) and a mixed linear model (MLM) were used to detect associations between QTL-markers and grain Cd concentrations using the TASSEL program (version 2.0; Bradbury et al., 2007). A significance threshold of 0.01 was used.

### Statistical analysis

Basic statistical analyses, such as Student's *t*-test, linear regression and two-way ANOVA, were performed mainly with Microsoft Excel 2010. Based on the ANOVA results, the phenotypic variances were partitioned and different components were calculated. Hence, genotypic variations, soil Cd levels, and the interaction between them could be estimated. The proportion of each variation in phenotypic variations could describe the contribution (Var%) of genotype, soil Cd concentration, or their interaction. Two significance levels were applied (*P* < 0.01 and *P* < 0.05).

## Results

### Phenotypic variation and heritability of grain Cd accumulation in rice hybrids and inbred cultivars

**Figure 1:**
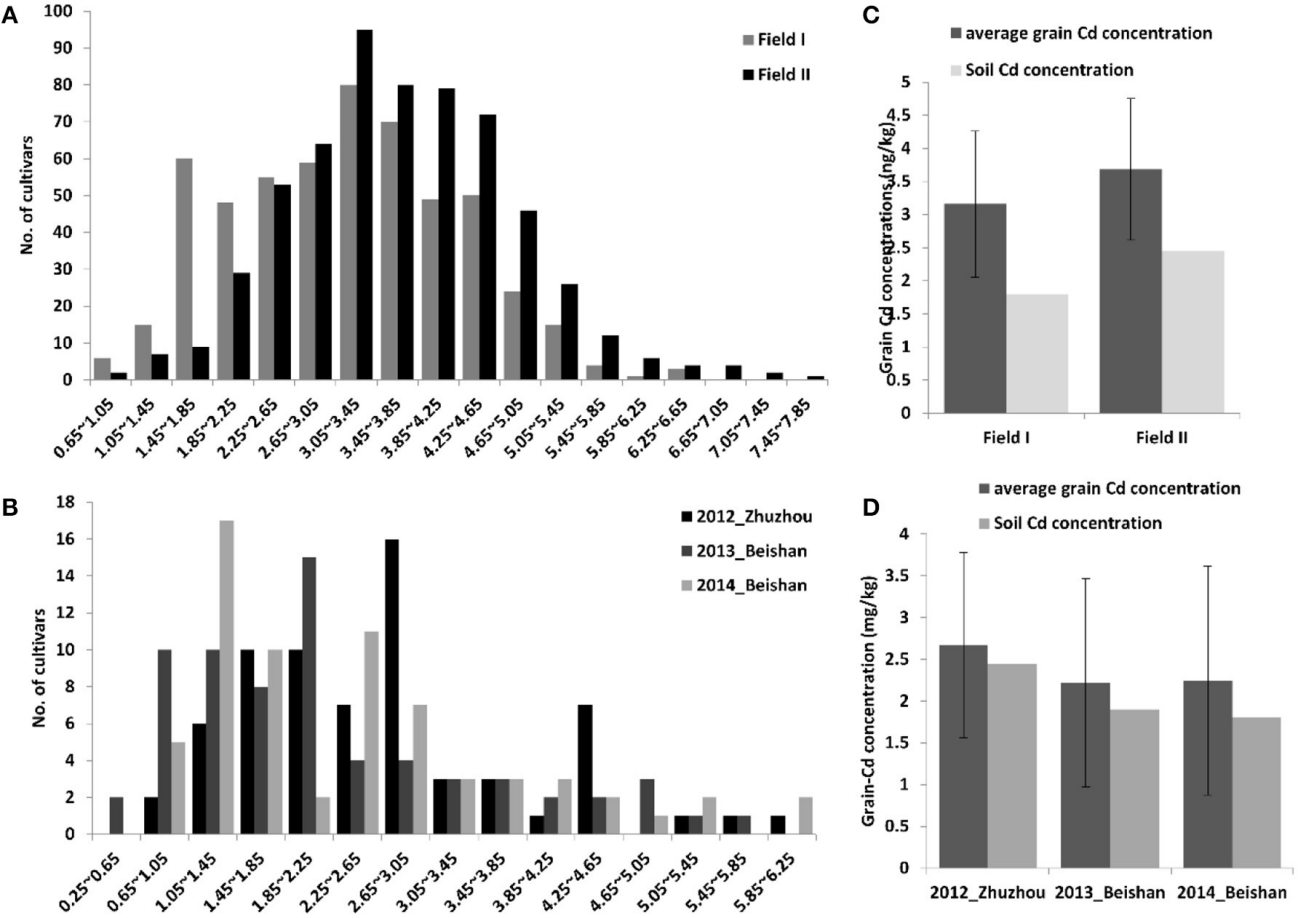
**Distribution of grain Cd accumulation and comparisons of the average grain Cd concentration in three different paddy fields**. The distributions of grain Cd accumulation in hybrid rice are shown in **(A)** and that in inbred rice cultivars in **(B)**. Comparisons of the average grain Cd levels and the soil Cd concentration in the different fields are shown for hybrid rice lines **(C)** and inbred rice cultivars **(D)**.

In this study, a total of 617 elite hybrid rice varieties collected from the middle and lower Yangtze River Valley in China were analyzed for their performance with respect to grain Cd accumulation. All of the hybrid varieties were planted for phenotyping the grain Cd concentrations in two paddy fields in 2014. The soil Cd concentrations were 1.80 mg/kg and 2.45 mg/kg in field I and field II with pH values of 5.4 and 5.8, respectively. As a quantitative trait, grain Cd concentration in the hybrids varied greatly (Figure 1A). The levels of Cd in the grain followed a normal distribution in both fields. In field I, grain Cd levels ranged from 0.67 to 6.58 mg/kg, with an average of 3.16 mg/kg and a median of 3.22 mg/kg. In field II, Cd levels ranged from 0.91 to 7.83 mg/kg, with an average of 3.68 mg/kg and a median of 3.63 mg/kg. In most cases, a cultivar grown in field II accumulated more Cd in the grain than it did in field I (*R*^2^ = 0.72, *P* = 4.0E-88), indicating that the grain Cd concentrations were greatly affected by the soil Cd levels (Figure 1C). The large variations in grain Cd accumulation observed in the different genotypes in the two fields suggested that genetic factors contributes to phenotypic divergence. To estimate the effects of genetic components in determining grain Cd accumulation, the phenotypic variations were divided into genotypic variations, soil Cd levels, and their interactions. By two-way ANOVA, all three parts had significant effects in determining grain Cd accumulations (*P* < 0.01; Supplemental Table 1). The heritability of grain Cd levels was detected as 50.8%, which predicted a stable heritability for grain Cd accumulation in hybrid rice.

**Figure 2:**
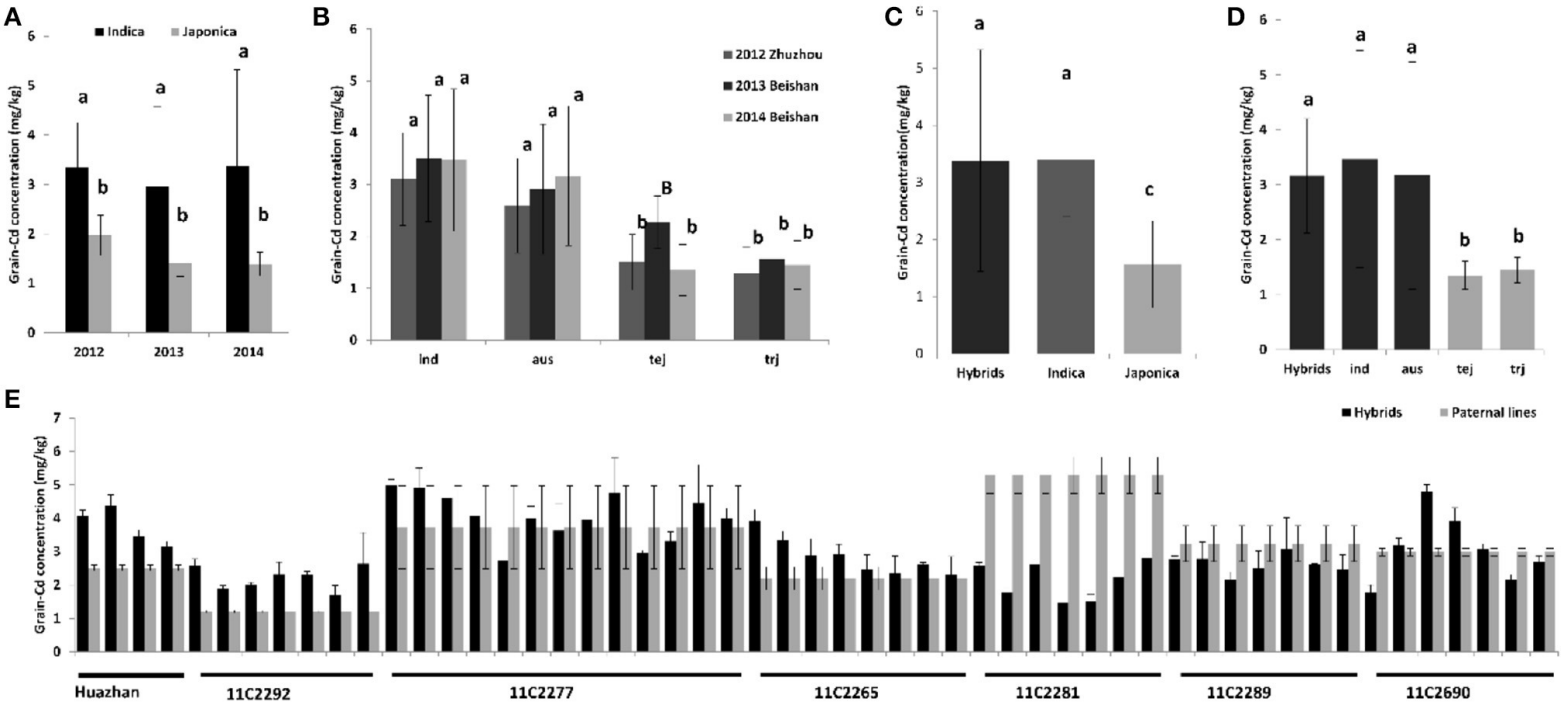
**Comparisons of grain Cd level among different subspecies, ecotypes, and varieties. (A)** Comparison of average grain Cd accumulation between the *indica* and *japonica* subspecies; **(B)** Comparison of average grain Cd accumulation among ecotypes. **(C)** Comparison of average grain Cd accumulation between the hybrids and two subspecies. **(D)** Comparison of average grain Cd accumulation between the hybrids and four ecotypes. **(E)** Comparisons of grain Cd concentration between each hybrid and its paternal inbred parental line. Different lower-case letters indicate significant differences at the 0.01 level, while same upper and lower case letters indicate significant differences at the 0.05 level.

We also analyzed the grain Cd performance in inbred rice cultivars independently. Sixty-eight cultivars were planted in three Cd polluted fields with varying Cd levels in three successive years (from 2012 to 2014; Figures 1B,D). The soil Cd contents of the three fields were 2.80, 1.90, and 1.80 mg/kg with pH values of 5.2, 5.0, and 5.4, respectively. Correspondingly, the average grain Cd concentrations for rice grown in these fields were 2.64, 2.29, and 2.43 mg/kg. Similar to the hybrids, genetic variations, soil-Cd levels, and their interactions are also key factors in determining phenotypic variation in the inbred cultivars (*P* < 0.01; Supplemental Table 1). The 68 cultivars were divided into two subspecies and five ecotypes; e.g. *ind, aus, aro, tej*, and *trj*, of which the first three belong to the *Indica* subspecies while the latter two are the *Japonica* subspecies (Garris et al., 2005). Significant inter-subspecies differences were detected (*P* < 0.01; Figures 2A,B). The grain Cd level of *Indica* rice were significantly higher than that of *Japonic* (*P* < 0.01). In contrast, the grain Cd accumulations did not differ by ecotype within a subspecies (*P* > 0.05). Without considering *aro* (only three varieties), the *ind* rice varieties accumulate Cd to levels similar to that of *aus* (*P* = 0.31), and *tej* rice accumulates Cd levels similar to that of *trj* (*P* = 0.30). Based on the genetic composition resolution by two-way ANOVA (Table 1), the *tej* ecotype performed the lowest genetic variations expansions of phenotypic variations (30.6%) and the highest soil-Cd expansions (as 40.0%). These results showed that grain Cd performance of the *tej* ecotype was the most easily influenced by soil Cd levels and that the *ind, aus*, and *trj* ecotypes were insensitive.

**Table 1:** **Variance analysis of grain Cd accumulation in different rice ecotypes by two-way ANOVA**.

**Ecotype**	**No. of cultivars**	**Components**	**Sum of square**	**Mean of square**	***F*-value**	**σ^2^**	**Var%**
*ind*	20	SCd	3.91	1.95	13.20^**^	0.05	2.6
		G	123.53	6.50	43.93^**^	1.06	61.0
		G × SCd	42.40	1.12	7.54^**^	0.48	27.9
*aus*	8	SCd	2.68	1.34	4.40^*^	0.07	2.5
		G	41.12	5.87	19.31^**^	0.93	35.4
		G × SCd	26.45	1.89	6.21^**^	1.33	50.6
*tej*	16	SCd	15.58	7.79	44.32^**^	0.24	40.0
		G	16.41	1.09	6.23^**^	0.18	30.6
		G × SCd	8.57	0.29	1.63	–	–
*trj*	12	SCd	0.90	0.45	12.99^**^	0.02	5.8
		G	13.48	1.23	35.39^**^	0.20	66.8
		G × SCd	3.83	0.17	5.03^**^	0.05	15.7
*aro*	3	SCd	3.13	1.57	13.33^**^	0.24	6.2
		G	38.85	19.43	165.47^**^	3.22	82.1
		G × SCd	4.60	1.15	9.79^**^	0.34	8.8
*mix*	9	SCd	1.08	0.54	1.77	–	–
		G	38.09	4.76	15.68^**^	0.74	71.0
		G × SCd	6.21	0.39	1.28	–	–

“G” represents the genotypic variations and “SCd” represents the soil Cd concentrations in three different paddy fields. “Var%” represents the expansion of different parts on determining the phenotypic variations. ^*^ and ^**^ indicate significant differences at P < 0.05 and P < 0.01, respectively.

We then compared Cd accumulation in the hybrids with the inbred cultivars of the different subspecies or different ecotypes (Figures 2C,D). All the hybrid combinations were phylogenetically grouped into the *Indica* subspecies in our study (see below). No significant differences in Cd levels were found among the hybrids, *ind*, and *aus* ecotypes of the inbred lines (*P* > 0.05). On the contrary, the *tej* and *trj* ecotypes accumulate significantly less Cd than do the *ind, aus*, or the hybrids (*P* < 0.01). For most hybrid combinations, the paternal line is usually an elite inbred variety. We therefore also compared the Cd accumulation in the hybrids with their paternal lines (Figure 2E). We found that variation in grain Cd levels between the hybrids and their paternal inbred lines existed, but no uniform pattern was observed. Hybrids accumulate more, less, or similar grain Cd levels to their paternal lines in a case-dependent manner, which agreed with our previous study (Yao et al., 2015; data not shown). In summary, our data indicated the hybrids do not accumulate more Cd than do the inbred cultivars or their paternal inbred lines.

### Relationship of grain Cd with heading date and other essential elements

Variation in heading date was present in our hybrid population (Supplemental Figure 1). Rice with a later heading date would be exposed to Cd for a longer time. However, there is no agreement about the relationship between heading date and grain Cd content (Ishikawa et al., 2005, 2010; Liu et al., 2005). To evaluate whether our observation of differences in grain Cd accumulation are determined by heading date, we investigated the relationship between grain Cd concentrations and heading date in hybrid rice. The rice hybrids could be divided into two subgroups: an early maturing group (< 75 days), and a later maturating group (≥75 days), based on the heading date. The average grain Cd level of hybrids in the early maturing group was lower than it was in hybrids in the later maturing group (*P* < 0.01). Linear regression analysis revealed that there was no significant correlation between grain-Cd with heading date in later maturing varieties (*P* > 0.05; Supplemental Figure 1B). Although a statistically significant positive correlation was detected for the early-maturing varieties (*P* < 0.01; *R*^2^ = 0.21; Supplemental Figure 1A), the low correlation coefficient (*R*^2^ < 0.3) in linear regression analysis indicated that the positive correlation was meaningless. If both groups were taken into account together, a significant positive correlation, but with *R*2 of 0.13, between grain Cd concentration and heading date was detected, suggesting the correlation was quite weak (Supplemental Figure 1C). Taking together, we did not detected a strong correlation between grain-Cd concentration and heading date, suggesting that the breeding of rice with low grain Cd levels could not be subjected to the limitation of heading date.

As a nonessential toxic heavy metal, the absorption and translocation of Cd always accompanies other essential elements in plants (Clemens, 2006; Clemens et al., 2013). In this study, by evaluating the relationships between Cd and four other essential elements, Fe, Mn, Cu, and Zn, we did not detect any significant correlation between Cd and any of the four essential metals (*P* > 0.05; Supplemental Table 2). Thus, the natural variation in grain Cd levels is independent of the accumulation of the other four essential microelements in this study, suggesting that it is possible to breed low grain-Cd hybrid rice without compromising the levels of the other micronutrients.

### Population structure analysis reveals that the hybrids belong to the *Indica* subspecies

**Figure 3:**
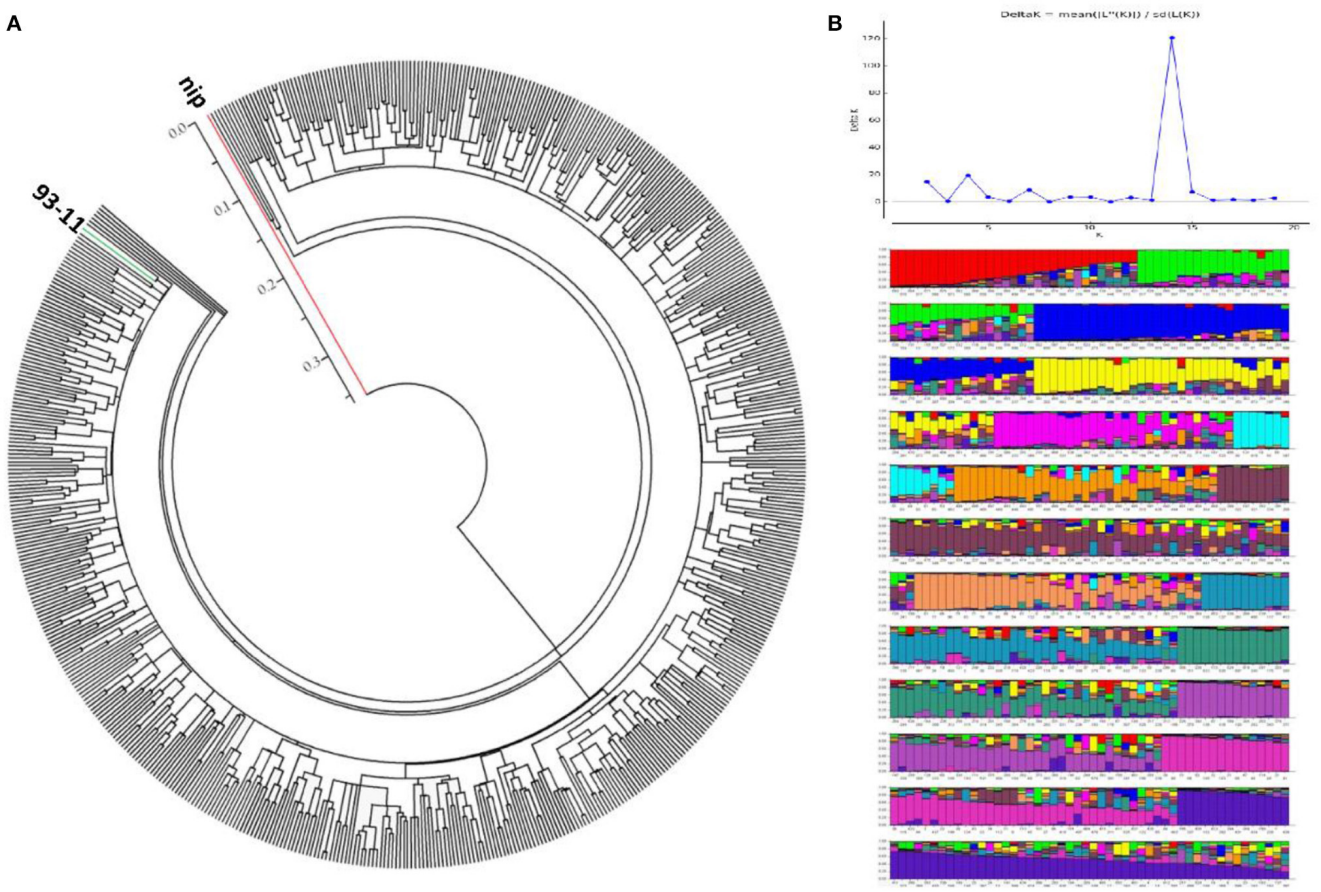
**Genetic structure of rice hybrids. (A)** Neighbor-joining tree showing the phylogenetic relationship of each hybrid rice out of 619 varieties drawn from 36 whole-genomic markers, including the two varieties, Nipponbare (a classical *Japonica* variety, red line), and 93-11 (a classical *Indica* variety, green line). **(B)** Population structure analysis of 617 rice hybrids at *K* = 14, as determined by STRUCTURE (version 2.3.4). Determination of *K*-value was followed the reporter of Evanno's study (Evanno et al., 2005) and genetated by STUCTURE Harvester (http://taylor0.biology.ucla.edu/structureHarvester/#).

Because the grain Cd accumulation in the hybrids was found to be similar to that of the *Indica* inbred cultivars, we determined the genetic backgrounds of the tested hybrids. The hybrids were genotyped with 36 molecular genetic markers covering the 12 chromosomes, including 22 SSR markers and 14 In/Del markers (Supplemental Table 3; Supplemental Data Sheet 1). Based on the genotyping results, 2–10 alleles were detected for each of the markers (an average of 5.17 alleles per marker), which indicated an appropriate level of genetic variation for population analysis. Using the marker genotypes of two typical cultivars, 93-11 and Nipponbare, as the references for the *Indica* and *Japonica* subspecies, respectively, a neighbor-joining (NJ) tree was generated using PowerMarker (Ver. 3.25) and MEGA (Ver. 5.0). There were no obvious population differentiations in the hybrid varieties, and all the hybrids grouped into the *Indica* subspecies (Figure 3A). Additionally, a STRUCTURE analysis produced the highest log-likelihood scores when the population number was set at 14, indicating that most of the hybrids could be classified into 14 sub-groups (Figure 3B). Intriguingly, combined with the geographic data from where the hybrids were developed, an ambiguous geographic distribution could be seen, in which some hybrid varieties with similar geographic origins tended to be clustered into the same clades (Supplemental Figure 2). For example, the hybrids produced from Zhejiang province were mainly clustered into one sub-group, while the hybrids from Fujian province were assigned with those from Jiangxi province. There were also exceptions; for example, hybrids from Hunan province were mainly dispersed among six sub-groups. Nevertheless, with the ambiguous geographic distribution, all the hybrids were still found to belong to the *Indica* subspecies (Figure 3A). In summary, on the basis of population structure analysis, all of the rice hybrids investigated in this study have a single *Indica* genetic background.

### Association analysis between Cd-related QTLs with grain-Cd concentrations in hybrids

Many QTLs relating to Cd in rice have been detected in different genetic populations. To determine whether these Cd-related QTLs regulate grain Cd accumulation in rice hybrids, we developed an association analysis of 14 related Cd-QTLs with grain Cd accumulation (Ueno et al., 2009, 2010; Abe et al., 2013; Yan et al., 2013; Zhang et al., 2013; Supplemental Figure 3; Supplemental Table 4). Based on previous reports, a total of 24 markers linked with these QTLs were designed from the Gramene database (http://www.gramene.org/) and used to characterize the genetic polymorphisms of the hybrid varieties. The GLM and the MLM were both used in our association analyses.

In field I, six loci showed significant associations under the GLM model and eight showed significance under the MLM model (*P* < 0.01). Specifically, the MLM model incorporated both a Q matrix (derived from STRUCTURE) and a Kinship matrix (to correct for the influence of population structure), which could be considered to control false positives. Together, five loci were detected in both models on four chromosomes; Chr 1, Chr 3, Chr 7, and Chr 11, (*P* < 0.01; Table 2). In field II, eight loci showed significant associations under the GLM model, and five loci were less significant under the MLM model (*P* < 0.01). Of these, three loci were detected in both models on chromosomes 7 and 11 at *P* < 0.01 (Table 2), and two significant loci on Chr 3 were also detected at *P* < 0.05. Taking all associated loci and both fields and models into consideration, a total of four loci were found to be consistently associated with variation in grain Cd levels (Supplemental Figure 3). Two loci, RM8006 on Chr 7 and RM6105 on Chr 11 showed extremely significant associations (*P* < 0.01), and another two loci, RM132 and RM16153 on chromosome 3, were detected at *P* < 0.01 in one field condition but at *P* < 0.05 in the other one.

**Table 2:** **Association analysis of 14 Cd-QTLs with grain Cd concentrations using two different models in different fields in rice hybrids**.

**ID**	**QTL**	**Chr**	**MLM model**				**GLM model**			
			**Field I**		**Field II**		**Field I**		**Field II**	
			**F_Marker**	**p_Marker**	**F_Marker**	**p_Marker**	**F_Marker**	**p_Marker**	**F_Marker**	**p_Marker**
1	RM6840	Chr.1	5.468^**^	0.004	1.196	0.303	16.360^**^	1.249E-07	6.091^**^	0.002
2	RM5465	Chr.2	5.264^*^	0.022	0.639	0.424	5.072^*^	0.0247	0.637	0.425
3	RM14095	Chr.2	2.151	0.143	0.078	0.780	5.668^*^	0.0176	0.458	0.498
4	RM166	Chr.2	0.424	0.655	3.229^*^	0.040	0.201	0.818	4.055	0.018
5	RM5378	Chr.2	0.833	0.436	2.175	0.114	1.468	0.2312	2.927	0.054
6	*RM132*	Chr.3	9.703^**^	0.002	5.310^*^	0.021	35.926^**^	3.684E-09	19.277^**^	1.361E-05
7	RM545	Chr.3	0.887	0.347	1.401	0.237	0.999	0.318	2.094	0.148
8	RM1338	Chr.3	0.748	0.387	1.599	0.206	0.499	0.481	1.461	0.227
9	RM1350	Chr.3	8.481^**^	0.004	\	\	5.196	0.023	2.666	0.103
10	*RM16153*	Chr.3	11.568^**^	7.31E-4	5.284^*^	0.022	12.721^**^	4.003E-4	5.655^*^	0.017
11	RM3295	Chr.5	1.680	0.1955	3.513	0.061	2.695	0.101	5.233^*^	0.022
12	RM4743	Chr.5	0.941	0.3782	1.874	0.342	1.114	0.294	2.145	0.089
13	RM21238	Chr.7	0.038	0.8458	2.422	0.120	4.059	0.044	7.298^**^	0.007
14	**RM8006**	Chr.7	7.916^**^	0.0051	8.791^**^	0.003	6.541^**^	0.001	8.694^**^	0.003
15	RM7153	Chr.7	7.387^**^	0.0068	7.915^**^	0.005	0.155	0.694	0.656	0.418
16	RM248	Chr.7	0.529	0.4673	0.451	0.502	1.018	0.313	0.095	0.757
17	RM149	Chr.9	2.937	0.0875	1.438	0.231	5.466	0.019	8.971^**^	0.002
18	RM1328	Chr.9	11.142^**^	9.042E-4	10.061^**^	0.001	1.198	0.274	0.428	0.513
19	RM219	Chr.9	\	\	1.781	0.149	1.742	0.157	1.622	0.183
20	RM215	Chr.9	1.121	0.326	3.239^*^	0.04	0.224	0.799	2.789	0.062
21	RM286	Chr.11	2.534	0.112	3.863	0.049	6.706	0.010	6.852^**^	0.009
22	RM4B	Chr.11	0.964	0.327	0.253	0.615	0.450	0.503	0.066	0.797
23	**RM6105**	Chr.11	5.582^**^	0.008	10.856^**^	0.001	12.262^**^	4.976E-4	14.316^**^	1.711E-4
24	RM6623	Chr.11	3.388	0.066	7.038^**^	0.008	15.258^**^	1.048E-4	17.596^**^	3.172E-05

* *P < 0.05*,

** *P < 0.01*;

*Bold font and italics indicate marker associations significant at P < 0.01 and P < 0.05, respectively, in both field and models*.

Of all the four marker loci, the low-Cd alleles have a low distribution in the hybrid population (Supplemental Figure 4). As mentioned above, the early maturating group (< 75 days) accumulated less grain Cd than did the late-maturing group (≥75 days) on average (*P* < 0.01). Correspondingly, the average low-Cd allelic distribution for the four associated loci in the early maturating group was higher (13.4%) than in the late maturing group (6.8%; Supplemental Figure 5). Therefore, the distribution of the low-Cd alleles from the four associated loci might be a causal factor for the differences in grain Cd concentrations between the two groups. This result could also explain the significant positive correlation between grain Cd concentration and heading date (Supplemental Figure 2), which might be due to the higher distribution of the low Cd alleles in the early maturing group than those in the later maturing group.

### *Indica-japonica* differentiation analysis for the four loci and performance of associated loci in hybrid crosses

**Figure 4:**
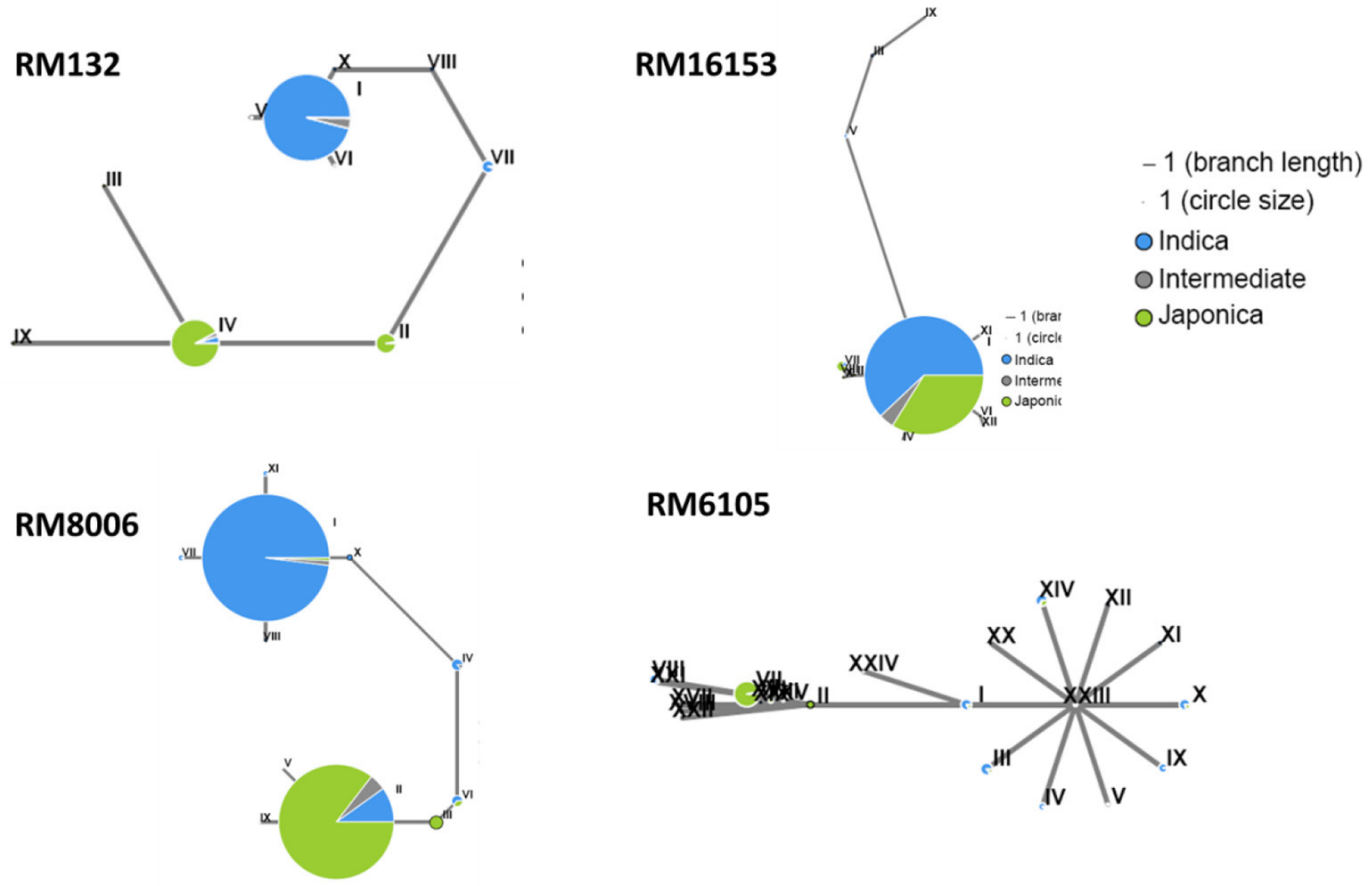
**Haplotype analysis of the genomic regions (~100 kb) flanking four associated markers**. The drafts were generated with 44 SNPs flanking each marker by using the analysis tool provide by RiceVarMap (http://ricevarmap.ncpgr.cn/hap_net/); green and blue represent the *japonica* and *indica* subspecies, respectively, and gray represents the intermediate.

In this study, we found that subspecies difference is an important factor with respect to grain Cd concentrations in a population of 68 cultivars (Figure 2A). Although there was no obvious population differentiation detected in the hybrid varieties (Figure 3A), we suspected that the subspecies differentiation of the associated loci might be responsible for the grain-Cd differences in the hybrids. Hence, to test our assumption, we investigated the *Indica*-*Japonica* differentiation for the four associated loci based on the RiceVarMap database (http://ricevarmap.ncpgr.cn/), which is a database of rice genomic variations (Zhao et al., 2015). For each associated locus, a total of 45 SNPs randomly distributed across a genomic region of 100 kb covering the associated locus were retrieved from the database. Then, based on the SNPs for each locus, we performed the haplotype network analysis in RiceVarMap (http://ricevarmap.ncpgr.cn/hap_net/; Supplemental Table 5). We identified 10, 12, 13, and 24 haplotype groups that were assembled in accordance with SNPs in the genomic regions flanking loci RM132, RM16153, RM8006, and RM6105, respectively. In support of our assumption, obvious *Indica*-*Japonica* differentiation was observed in the genomic regions around RM132, RM8006, and RM6105, but not for RM16153 (Figure 4).

**Figure 5:**
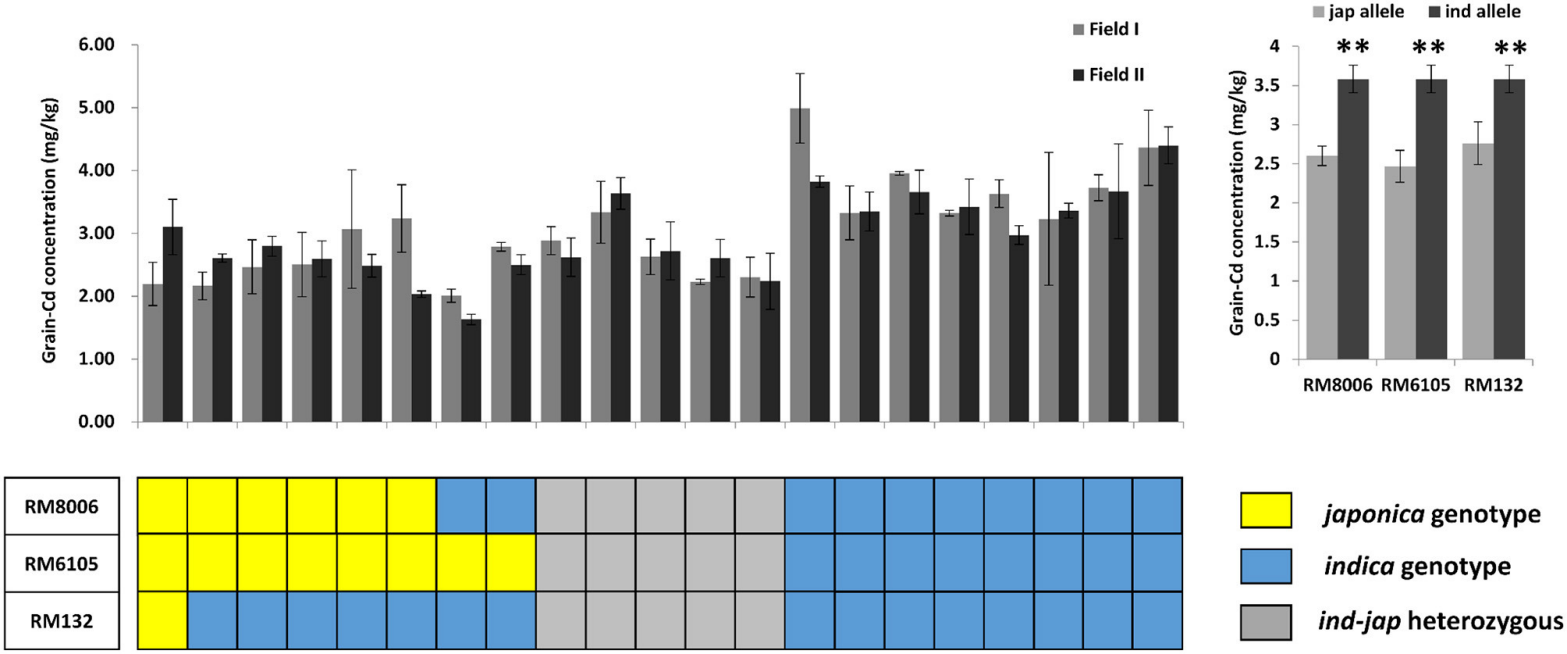
**Genetic effects of the three *indica*-*japonica* differentiation loci on grain Cd concentrations in the 21 hybrid rice combinations**. Nipponbare and 93-11 were used to distinguish the *japonica* and *indica* alleles; *japonica* alleles are shown in yellow, *indica* alleles are shown in blue, and the gray blocks represent the *indica*-*japonica* heterozygote. ^**^ indicates significance at *P* < 0.01.

To further confirm the above result, we made a population of 21 hybrid crosses and tested the genetic effects of the four loci in a breeding application. 93-11 and Nipponbare were used to represent the *Indica*-*Japonica* diversity. The *Indica*-*Japonica* variations were genotyped for RM132, RM8006, and RM6105 in all the 21 hybrid crosses; RM16153 was not used because there are no allelic variations in the 21 hybrid crosses (Figure 5). In total, the hybrids with the *Japonica* alleles accumulated less Cd than those with the *Indica* alleles. The *Japonica* alleles for RM6105 and RM8006 significantly reduced the grain Cd concentration in either the homozygous or heterozygous state (*P* < 0.01). Because only one line was homozygous for the *Japonica* allele of RM132, it was omitted from the statistical analysis. Nevertheless, the *Indica*-*Japonica* heterozygosis at RM132 was found to reduce the grain Cd concentration significantly (*P* < 0.01). Although the genetic backgrounds of the 21 hybrid combinations were not clear, the *Indica*-*Japonica* diversity at the three loci could reduce the grain Cd concentrations. Thus, the three linked loci, RM132, RM8006, and RM6105, might be applied to MAS for low-Cd hybrid breeding programs. These results confirmed our assumption that genetic diversity, especially the *Indica*-*Japonica* differentiation, might play a major role in variation in grain Cd accumulation.

## Discussion

Traditionally, the people in southern Asia, where hybrid rice originated and now dominates rice production, have preferred to consume more rice products. The characteristics of high yield heterosis and efficient water and fertilizer utilization of rice hybrids raises concerns about the over-accumulation of Cd in the grains. Based on the 2001 report of the national nutrition survey in China, a high risk of Cd intake is already present in this region (Li et al., 2005). It is imperative to reduce grain Cd accumulation in rice, especially in high-yielding hybrid rice, and it is a prerequisite to have comprehensive knowledge of the underlying genetic mechanism(s) that control Cd accumulation.

It is generally accepted that there is considerable natural variation in Cd accumulation present among different rice varieties (Arao and Ae, 2003; He et al., 2006). Previous studies have indicated that *Indica* rice varieties usually accumulate more Cd than do *Japonica* rice varieties (Chaney et al., 2004; Hui et al., 2006). However, a consistent conclusion has not been obtained by comparing Cd accumulation between two cultivar types: hybrid rice and traditional inbred cultivars (Liu et al., 2005; Gong et al., 2006; Gong and Pan, 2006; Yao et al., 2015). Hybrid rice is distinct from traditional cultivated rice through its breeding process and the fact that rice hybrids are usually developed by crossing two parental inbred lines. Consequently, the grain Cd levels of rice hybrids could be decided by the genetic backgrounds of the parental lines. Our previous report showed that additive parental effects dominated the grain Cd performance in hybrid rice with no obvious heterosis present (Yao et al., 2015). By comparing grain Cd accumulation between 617 *Indica* rice hybrids and 68 cultivars of different ecotypes in this study, we discovered that hybrid rice did accumulate more grain Cd than did the *tej* and *trj* ecotypes, but not more than the *ind* or *aus* ecotypes (Figure 2D). A genetic background survey using genomic markers revealed that all of the hybrids tested in our study are *Indica* rice genetically (Figure 3A). Therefore, it is the genetic background but not the hybrid cultivar type that determines performance of hybrid combinations with respect to grain Cd accumulation. Based on this knowledge, it is not difficult to obtain a clear understanding of the discrepancy from previous studies. We surveyed the genetic background of the rice cultivars used in the previous studies in which it was declared that hybrid rice accumulated more Cd than do inbred lines (Gong et al., 2006; Gong and Pan, 2006). Most of the hybrids were *Indica* while the inbred lines belonged to the *Japonica* subspecies. Thus it was not unreasonable to conclude that the hybrids accumulated more grain Cd than did the inbred cultivars in that study. Taking the genetic background into consideration, Liu et al. (2005) found that the levels of Cd in *Japonica* varieties and *Japonica* hybrids were significantly lower than in *Indica* varieties and hybrids; and the difference in Cd concentrations in brown rice between *Japonica* varieties and hybrids, and between *Indica* varieties and hybrids were not significant. Our study suggests that it was not reasonable to make a conclusion on the comparison of Cd accumulation between hybrids and inbred cultivars without considering their genetic backgrounds; and that hybrid rice does not accumulate more grain Cd than do inbred lines.

Adequate assessment of the genetic effects on grain Cd accumulation is a prerequisite for reducing Cd levels in rice grain. In our study, the differences in grain Cd accumulation were determined by the genotypic diversity, the differences in soil Cd levels, and their interaction (*P* < 0.01). In our study a stable heritability for grain Cd accumulation was predicted in hybrids (as high as 50.8%). Analysis of population structure in the hybrids revealed no obvious population structure differentiation in our hybrid population, but showed that all of the hybrids belonged to the *Indica* subspecies (Figure 3A). Therefore, the results suggested that the single *Indica* background and the stable inheritance of grain Cd accumulation in the present hybrids, just like those in the *Indica* inbred cultivars, probably caused the high grain Cd accumulation in hybrid rice (Liu et al., 2005; Gong et al., 2006). Two-way ANOVA revealed that the *ind* and *trj* ecotypes, rather than *aus* and *tej*, had stable grain Cd accumulation performance (Table 1). Thus, it is important to screen and identify stable, low-Cd-accumulating cultivars from the *ind* and *trj* ecotypes, and not the *aus* and *tej* ecotypes, in order to enhance the efficiency of breeding for low grain Cd levels.

Although all the tested hybrids are *Indica* rice genetically, the levels of Cd in the grain varied by more than 10-fold, ranging from 0.67 to 7.83 mg/kg (Figure 1A). Thus it should be possible to breed high yielding hybrid rice lines with low grain Cd accumulation. Generally, as a quantitative trait, grain Cd accumulation cannot be selected directly (Xing and Zhang, 2010; Rao et al., 2014). The identification of QTLs/QTL-genes and using MAS will benefit rice breeding programs directed toward low grain Cd accumulation. In recent years, many QTLs for Cd absorption, transportation, and accumulation have been identified in rice (Ueno et al., 2009, 2010; Ishikawa et al., 2010; Yan et al., 2013; Zhang et al., 2013). In our study, four QTLs out of 14 Cd-related QTLs showed significant associations with grain Cd concentration in the hybrids, and are distributed on chromosomes 3, 7, and 11. Using haplotype analysis, three genomic regions linked with each marker (RM132, RM8006, and RM6105) showed obvious *Indica*-*Japonica* differentiation (Figure 4). This result suggested that alleles derived from *Japonica* might reduce grain Cd accumulation in the hybrids. Heterosis of inter-subspecies hybrids are extensively explored in current hybrid rice breeding, and an extra benefit of low-Cd rice would result from exploration of hybrid heterosis between *indica* and *japonica* cultivars. In addition, although there was no *Indica*-*Japonica* differentiation in the region surrounding RM16153 (Figure 4), it seems that the allelic variation within the *Indica* subspecies also contributes to the variations in grain Cd levels. In summary, the genetic diversity, especially the differentiation between the *Indica* and *Japonica* subspecies, of QTL loci for Cd accumulation will be valuable in reducing grain Cd levels in the hybrids.

In modern breeding practices, phenotypic selection is correlated with the strong alleles of major genes (Huang et al., 2015). The alleles with stable effects on breeding characteristics are useful. In our previous study, we found that the additive parental effect was dominant in grain Cd accumulation in the hybrids (Yao et al., 2015). In the present study, the average distribution of the effective alleles for the four QTLs was 8.3% (5.6% for RM132, 3.9% for RM16153, 10.1% for RM8006, and 13.4% for RM6105), which indicates a great potential for hybrid improvement (Supplemental Figure 4). Thus, pyramiding multiple efficient QTLs or alleles could be useful in reducing the grain Cd accumulation in the hybrids. In the last decade, several genes involved in Cd accumulation in rice have been identified, such as *OsIRT1, OsNramp1, OsHMA3, OsNramp5, OsHMA2, OsLCD* and *OsLCT1* (Ueno et al., 2010; Shimo et al., 2011; Takahashi et al., 2011; Uraguchi et al., 2011; Ishikawa et al., 2012). However, the natural variation and effects on grain Cd accumulation are still to be determined for these genes. One of the QTLs on chr 7 that we detected to be associated with Cd variation in the hybrids overlaps with a region where four Cd-related genes mapped, such as *OsHMA3, OsNramp1, OsNramp5*, and *OsZIP8* (Ueno et al., 2009, 2010; Takahashi et al., 2011; Ishikawa et al., 2012; Sasaki et al., 2012). After identifying the effects of these genes on natural variations in grain Cd accumulation, the superior alleles, if they do not compromise other agricultural traits, might be potential genetic tools for facilitating the breeding for low grain Cd levels in high yielding rice hybrids.

## Author contributions

CC and LS designed the research work, annotated the data and drafted the manuscript. YY, ZH, AL, WT, GX prepared plant populations. LS, XX, YJ, QZ, FY, JZ, LC, GZ, JW, DH performed the experiments.

### Conflict of interest statement

The authors declare that the research was conducted in the absence of any commercial or financial relationships that could be construed as a potential conflict of interest.
