# Supplementary material for: Genetic Diversity, Rather than Cultivar Type, Determines Relative Grain Cd Accumulation in Hybrid Rice
Source: Front Plant Sci. 2016 Sep 21;7:1407. doi: 10.3389/fpls.2016.01407 (PMC5030296; doi:10.3389/fpls.2016.01407)
Supplement: Supplementary file 1 [file Table1.DOCX]

**Supplemental Table 1. Analysis of variation in grain Cd levels in the hybrids and inbred cultivars by two-way ANOVA**

| types | Components | SS | MS | F |
| --- | --- | --- | --- | --- |
| Hybrids | Soil-Cd | 204.30 | 204.30 | 441.64** |
|  | Genetic variations | 3200.82 | 5.45 | 11.79** |
|  | Genetic variations× Soil-Cd | 572.76 | 0.98 | 2.11** |
|  | others | 1088.01 | 0.46 |  |
| Inbred culitvars | Soil-Cd | 13.64 | 6.82 | 39.62** |
|  | Genetic variations | 528.40 | 7.88 | 45.82** |
|  | Genetic variations× Soil-Cd | 105.70 | 0.79 | 4.58** |
|  | others | 35.11 | 0.17 |  |

* and ** indicate significant differences at *P*<0.05 and *P*<0.01, respectively.

**Supplemental Table 2. Correlation coefficients between the levels of Cd and other four essentials elements (Cu, Fe, Mn, and Zn) in hybrid rice grain**

|  |  | Grain-Cu | Grain-Fe | Grain-Mn | Grain-Zn |
| --- | --- | --- | --- | --- | --- |
| Field I | Grain-Cd | -0.054 | -0.038 | 0.193 | 0.002 |
| Field II |  | -0.056 | 0.013 | 0.15 | 0.136 |

* and ** indicate significant differences at *P*<0.05 and *P*<0.01, respectively.

**Supplemental Table 3. Genomic markers used in the analysis of population structure in hybrid rice**

| Marker | Chr | F | R |
| --- | --- | --- | --- |
| RM495 | 1 | AATCCAAGGTGCAGAGATGG | CAACGATGACGAACACAACC |
| RM1 | 1 | gcgaaaacacaatgcaaaaa | gcgttggttggacctgac |
| ID3282 | 1 | GACATCCTTCACCAACCGTT | GGTATAGGTAGAACCTATTGG |
| ID0220 | 2 | GGGATAGTAAGTGGTGTGACT | CTGAGCTAGGAAGGATCGAT |
| RM71 | 2 | ctagaggcgaaaacgagatg | gggtgggcgaggtaataatg |
| ID3325 | 2 | CGAGTATCCAAGGCTCAAGT | GCCACCCAAATCTGCTATCT |
| RM232 | 3 | ccggtatccttcgatattgc | ccgacttttcctcctgacg |
| RM282 | 3 | CTGTGTCGAAAGGCTGCAC | CAGTCCTGTGTTGCAGCAAG |
| RM85 | 3 | ccaaagatgaaacctggattg | gcacaaggtgagcagtcc |
| R4ID0714 | 4 | CCCGATATGTCTATGCTAGC | CCAGACCCCAAATCCCTTCT |
| RM307 | 4 | gtactaccgacctaccgttcac | ctgctatgcatgaactgctc |
| RM348 | 4 | GGAGCTTTGTTCTTGCGAAC | GGAGCTTTGTTCTTGCGAAC |
| RM413 | 5 | ggcgattcttggatgaagag | tccccaccaatcttgtcttc |
| RM598 | 5 | gaatcgcacacgtgatgaac | atgcgactgatcggtactcc |
| RM274 | 5 | cctcgcttatgagagcttcg | cttctccatcactcccatgg |
| R6ID0030 | 6 | GAGGATGGTTCAAAGCGAAA | CGACATCCCCATGTTCAGAT |
| R6ID1312 | 6 | GAGCACATGTTGGTGGGACT | GAGAACAACCCAGAGGATGA |
| R6ID2660 | 6 | GCCATGTGTCACGTCTAGTA | GGTACGGGAACCCACAGTT |
| RM5752 | 7 | CATCTCAACCTGCGAGGAAG | CTGAGAGAGACACACACAGCG |
| RM214 | 7 | CTGATGATAGAAACCTCTTCTC | AAGAACAGCTGACTTCACAA |
| R7ID2122 | 7 | GCCAACAGGTCATCAGCCAT | GTGCCAATAACTTGATTGAGC |
| RM25 | 8 | GGAAAGAATGATCTTTTCATGG | CTACCATCAAAACCAATGTTC |
| ID1243 | 8 | CAACCAGACAAGATCCTTGAG | CCGGAACAAAATCTGGATTAC |
| RM210 | 8 | TCACATTCGGTGGCATTG | CGAGGATGGTTGTTCACTTG |
| ID739 | 9 | GGTTTCGGTACCTTCTCACT | CAAGTCAGTGATGCATAAACC |
| R9ID1266 | 9 | GCGTCTCCGTGTTTGTTTGT | GAGCATTGATAGAGAGTGACG |
| RM215 | 9 | CAAAATGGAGCAGCAAGAGC | TGAGCACCTCCTTCTCTGTAG |
| R10ID0471 | 10 | CCTGTCGAACAGCTTAGGAA | GTGGACCTGCTCTATTACAG |
| RM2504 | 10 | TAACACAACAATAGCGTCAG | TAGGAAGAACTGAAGAAGCA |
| RM590 | 10 | catctccgctctccatgc | ggagttggggtcttgttcg |
| RM332 | 11 | GCGAAGGCGAAGGTGAAG | CATGAGTGATCTCACTCACCC |
| R11ID2445 | 11 | GTCATAGCAACGGAAAATGC | CATCCTGGGCTCTTGGATAA |
| RM144 | 11 | TGCCCTGGCGCAAATTTGATCC | GCTAGAGGAGATCAGATGGTAGTGCATG |
| RM19 | 12 | caaaaacagagcagatgac | ctcaagatggacgccaaga |
| R12ID1072 | 12 | GTTCTACGAGACAGCAATGG | CCACGCTGCTAATTCTGACT |
| RM270 | 12 | GGCCGTTGGTTCTAAAATC | TGCGCAGTATCATCGGCGAG |

**Supplemental Table 4. The linkage markers of the 14 Cd-QTL used in association analysis**

|  | F | R | location | references |
| --- | --- | --- | --- | --- |
| RM6840 | CGACTGGAAGAAGGGATCATGG | CACACTACCAAGACTCCGCTATGG | chr.1 | Zhang et al., 2014 |
| RM5465 | CAATGAAATTGGTGGCTCACTGG | TACTAGGTCGGCCCGCTTAGATAGC | Chr.2 | Ueno et al., 2009 |
| RM14095 | CGGCTTGTTTATGCTACCAGAGG | CGAGGAGACTAACCAAATTGATCG | Chr.2 | Ueno et al., 2009 |
| RM166 | GGTCCTGGGTCAATAATTGGGTTACC | TTGCTGCATGATCCTAAACCGG | Chr.2 | Zhang et al., 2014 |
| RM5378 | GCTCGGCTGCGTTCTACTAC | AGAAAGGAGGGAGCCGATAG | chr.2 | Ueno et al., 2009 |
| RM132 | CTTTCTCTCGCCTACGCCTTCC | TCGACGAGGTTGATCAGTAGGG | Chr.3 | Zhang et al., 2014 |
| RM545 | CCTTCCCTGAAAGTATTCGTTCTCC | GAGAACGTCTTCATTGGATGTTCC | Chr.3 | Yan et al., 2013 |
| RM1338 | TGCAAGTTGGACTTCAAAGAGG | TGGATTCCTTCTTCCTTTCTCTCC | Chr.3 | Yan et al., 2013 |
| RM1350 | AGGAACACCCAAGAGAGTCATGC | GCAAGAAAGCTCTGCTCCATGC | Chr.3 | Abe et al., 2013 |
| RM16153 | TGGTTGTGGTATAGCACGGTAAGC | TGACCCAAGGAGATACTAGGTTGC | Chr.3 | Abe et al., 2013 |
| RM3295 | AGACACGGCAAGGACAAAGC | CGTTCGGACTCCTTTGGATAGC | Chr.5 | Ueno et al., 2009 |
| RM4743 | CTGTTAGGCAGGCTAGTCTGAGAGC | GATGCTCGTGGTCTGAATTGG | Chr.5 | Ueno et al., 2009 |
| RM21238 | GAGCTTCTCCTCACCCATCACC | CTTCTGCAGAGGGTGTTCAACG | Chr.7 | Ueno et al., 2010 |
| RM8006 | AATTTGAGTAGGAGGTCGTAGCC | GATGAATGGTCCACATTACTCC | Chr.7 | Ueno et al., 2010 |
| RM7153 | AACCGATCAGCAACCATCCAAAGG | GTTGCACGGTGGATGACGTTGG | Chr.7 | Ueno et al., 2010 |
| RM248 | AGAGAGCAAGTTTGAAGCGAAGC | ACCAAGAGGGTAGCCTAGCATGG | Chr.7 | Abe et al., 2013 |
| RM149 | GGAAGCCTTTCCTCGTAACACG | GAACCTAGGCCGTGTTCTTTGC | chr.8 | Zhang et al., 2014 |
| RM1328 | GAATGGGATTAGACGATTTG | CCATGAGTGACATCAAAAGG | chr.9 | Yan et al., 2013 |
| RM219 | cgtcggatgatgtaaagcct | catatcggcattcgcctg | chr.9 | Yan et al., 2013 |
| RM215 | GAGCAGCAAGAGCAGCAGAGG | CATGCTCGACTTCAGAAGCTTGG | chr.9 | Zhang et al., 2014 |
| RM286 | CTGGCCTCTAGCTACAACCTTGC | AAACTCTCGCTGGATTCGATAGG | Chr.11 | Yan et al., 2013 |
| RM4B | CGTTGATTCGAAGGGTGTATCC | GAGGTCAGCACTGACGAGTTAAGC | Chr.11 | Yan et al., 2013 |
| RM6105 | ACGTCTTTCCAGCCACAATCACC | CGCTTCTGGAAGGGAGGAGAGG | Chr.11 | Ueno et al., 2009 |
| RM6623 | GACACACAAACACCTCACACACC | AGATTCTTGCGAGCGAGG | Chr.11 | Ueno et al., 2009 |

**Supplemental Table 5. Haplotype distribution of associated locus regions (~100 kb) from the RiceVarMap database**

| Locus | Haplotype Group | Ind | Jap | Inter |  | Locus | Haplotype Group | Ind | Jap | Inter |  | Locus | Haplotype Group | Ind | Jap | Inter |
| --- | --- | --- | --- | --- | --- | --- | --- | --- | --- | --- | --- | --- | --- | --- | --- | --- |
| RM132 | H1 | 641 | 2 | 24 |  | RM6105 | H1 | 65 | 12 | 0 |  | RM8006 | H1 | 72 | 1 | 1 |
|  | H2 | 2 | 149 | 2 |  |  | H2 | 0 | 48 | 0 |  |  | H2 | 45 | 405 | 22 |
|  | H3 | 0 | 18 | 0 |  |  | H3 | 74 | 11 | 1 |  |  | H3 | 0 | 51 | 0 |
|  | H4 | 18 | 337 | 9 |  |  | H4 | 47 | 4 | 2 |  |  | H4 | 309 | 3 | 3 |
|  | H5 | 16 | 0 | 1 |  |  | H5 | 12 | 5 | 1 |  |  | H5 | 11 | 0 | 0 |
|  | H6 | 10 | 0 | 1 |  |  | H6 | 0 | 21 | 0 |  |  | H6 | 38 | 0 | 7 |
|  | H7 | 91 | 0 | 3 |  |  | H7 | 2 | 176 | 3 |  |  | H7 | 21 | 0 | 1 |
|  | H8 | 25 | 0 | 0 |  |  | H8 | 64 | 2 | 0 |  |  | H8 | 33 | 0 | 1 |
|  | H9 | 0 | 16 | 0 |  |  | H9 | 61 | 0 | 1 |  |  | H9 | 13 | 0 | 0 |
|  | H10 | 23 | 0 | 0 |  |  | H10 | 62 | 14 | 1 |  |  | H10 | 4 | 6 | 1 |
|  |  |  |  |  |  |  | H11 | 18 | 0 | 0 |  |  | H11 | 27 | 0 | 0 |
|  |  |  |  |  |  |  | H12 | 13 | 0 | 0 |  |  | H12 | 18 | 0 | 0 |
| RM16153 | H1 | 654 | 357 | 43 |  |  | H13 | 2 | 34 | 0 |  |  | H13 | 13 | 0 | 1 |
|  | H2 | 0 | 19 | 0 |  |  | H14 | 57 | 22 | 5 |  |  |  |  |  |  |
|  | H3 | 22 | 0 | 0 |  |  | H15 | 21 | 1 | 0 |  |  |  |  |  |  |
|  | H4 | 9 | 5 | 0 |  |  | H16 | 0 | 16 | 0 |  |  |  |  |  |  |
|  | H5 | 37 | 0 | 2 |  |  | H17 | 13 | 1 | 0 |  |  |  |  |  |  |
|  | H6 | 0 | 14 | 0 |  |  | H18 | 16 | 0 | 0 |  |  |  |  |  |  |
|  | H7 | 17 | 73 | 0 |  |  | H19 | 16 | 0 | 0 |  |  |  |  |  |  |
|  | H8 | 8 | 3 | 1 |  |  | H20 | 12 | 0 | 0 |  |  |  |  |  |  |
|  | H9 | 11 | 0 | 0 |  |  | H21 | 10 | 1 | 0 |  |  |  |  |  |  |
|  | H10 | 0 | 17 | 0 |  |  | H22 | 12 | 0 | 0 |  |  |  |  |  |  |
|  | H11 | 5 | 6 | 0 |  |  | H23 | 3 | 22 | 3 |  |  |  |  |  |  |
|  | H12 | 0 | 11 | 0 |  |  | H24 | 1 | 8 | 4 |  |  |  |  |  |  |


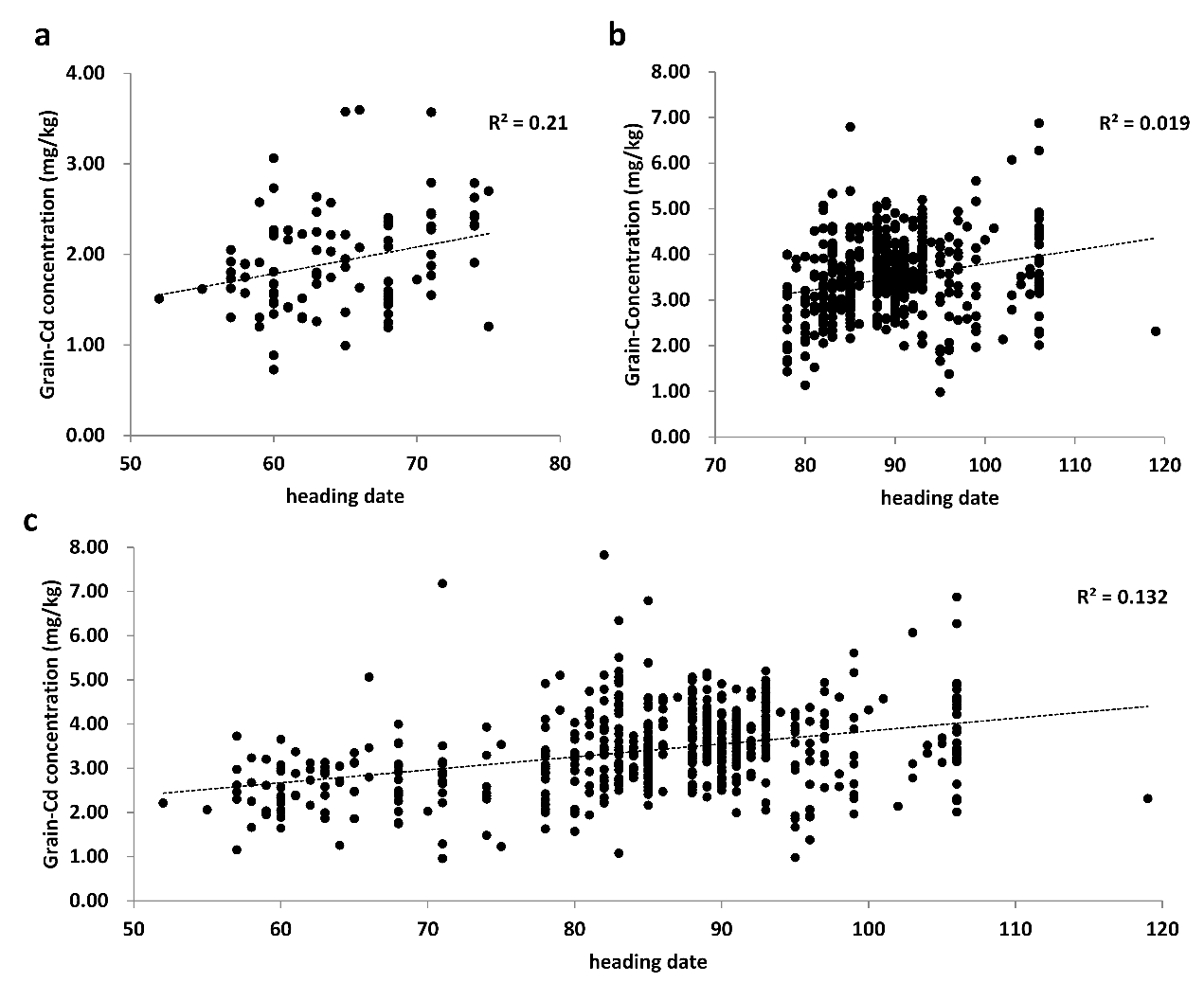


**Supplemental Figure 1. Regression analysis shows that grain Cd levels are independent of heading date in hybrid rice**

a is regression analysis of grain-Cd concentration vs. heading date in the early maturating group (<75 days); b is regression analysis of grain-Cd concentration vs. heading date in the early maturating group (≥75d); c is regression analysis of grain-Cd concentration vs. heading date in all the testing hybrids.

**
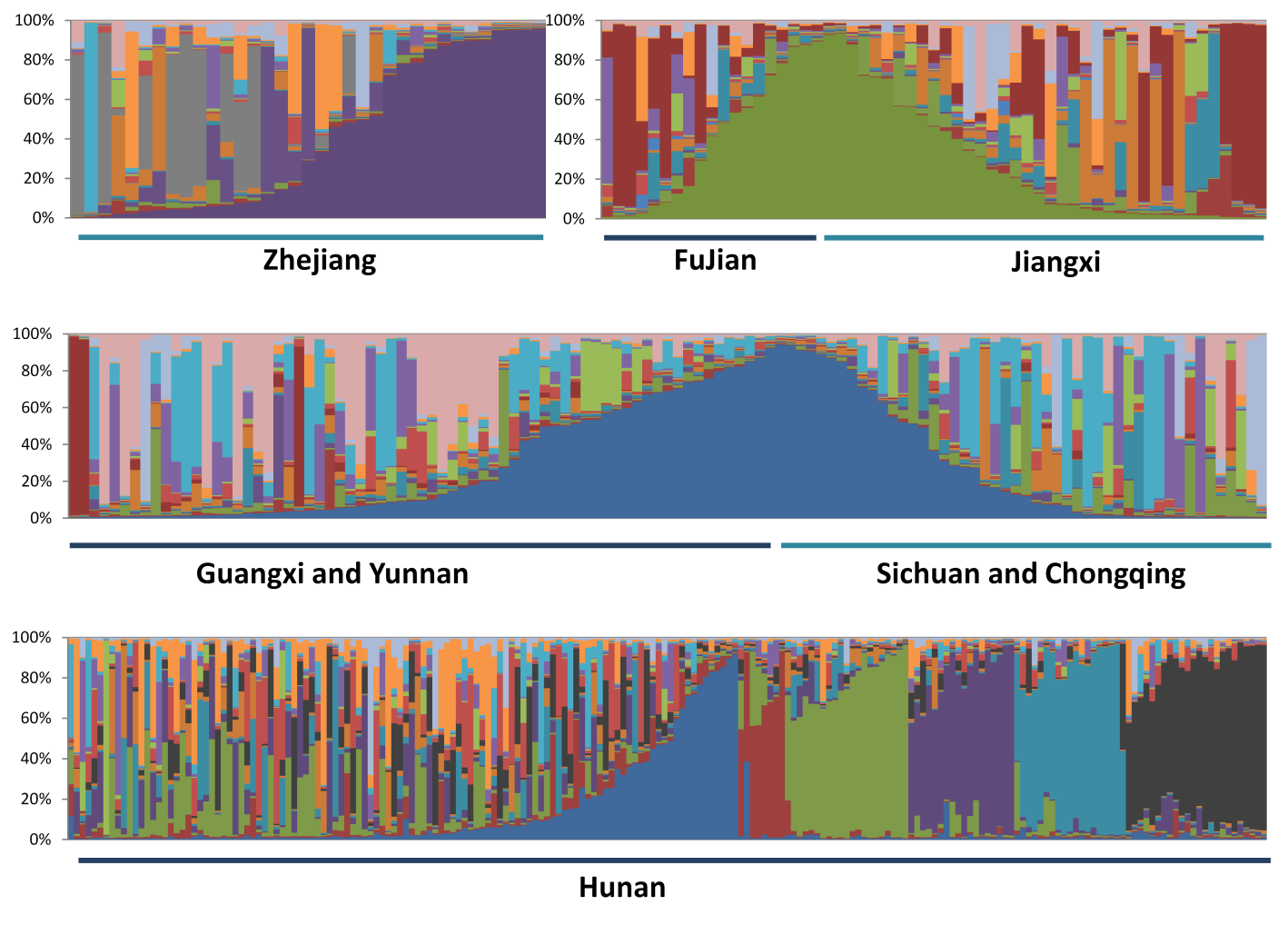
**

**Supplemental Figure 2. Ambiguous geographical distribution by population structure analysis**


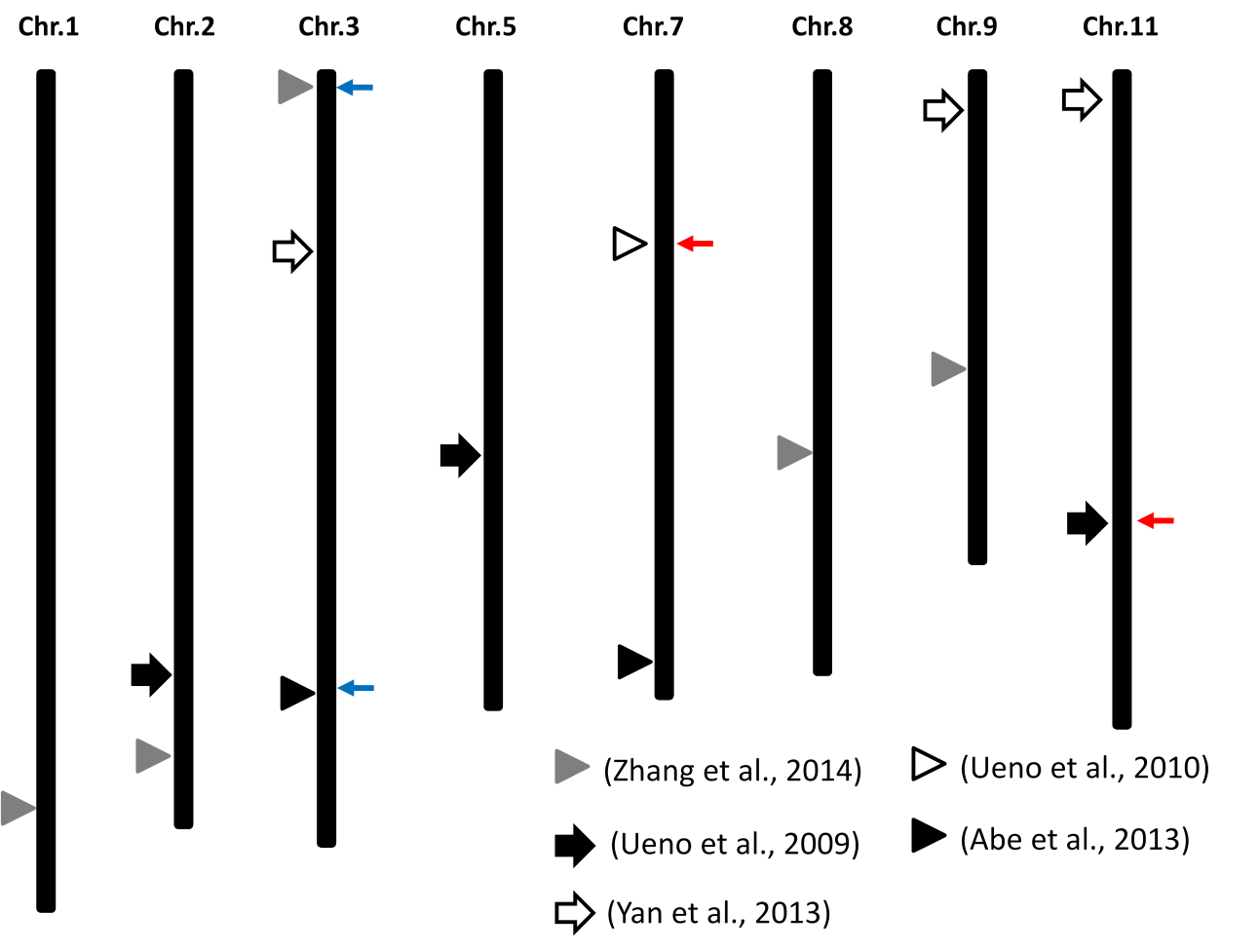


**Supplemental Figure 3. Distribution of 14 related Cd-QTLs and the loci associated with grain Cd accumulation in hybrid rice**

The red arrows respects the extremely significantly loci out of the 14 Cd-QTLs; the blue arrows respects the less significantly loci.

**
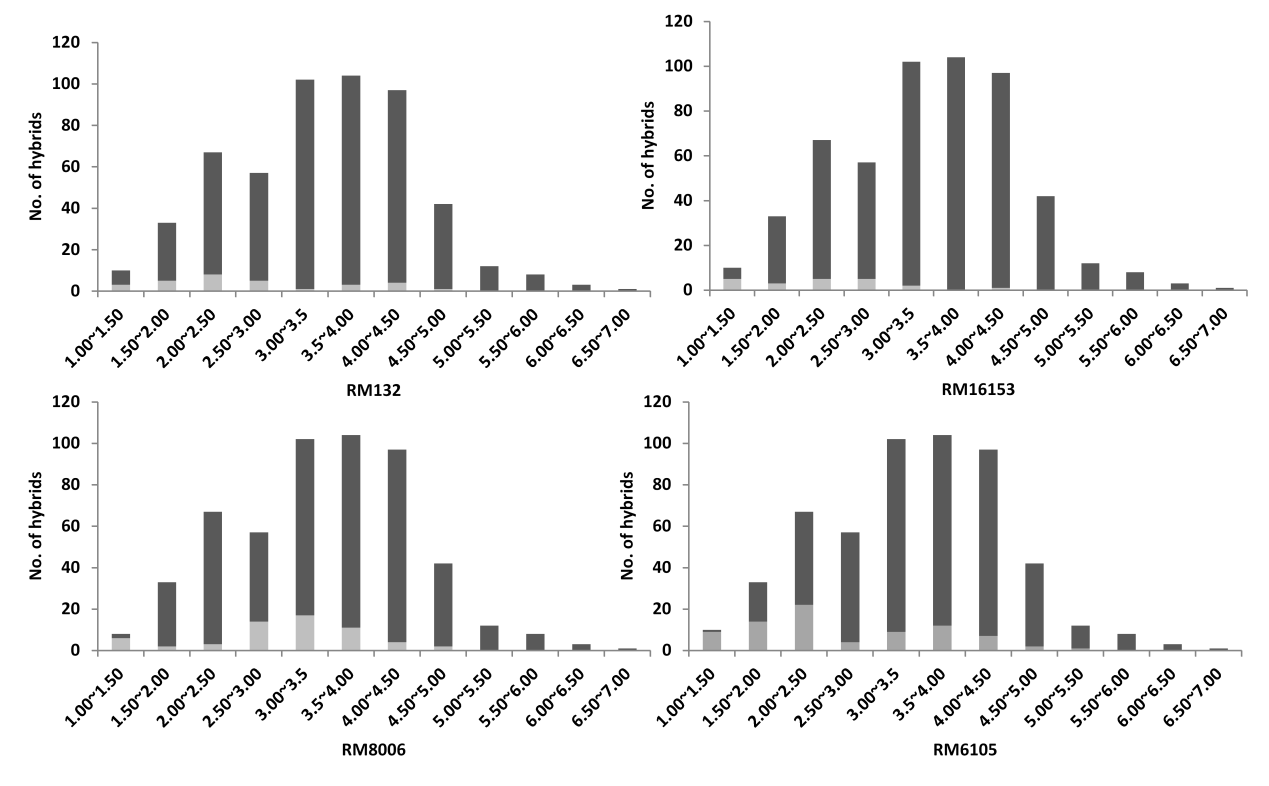
**

**Supplemental Figure 4. Distribution of four Cd-associated loci in hybrid rice**

**
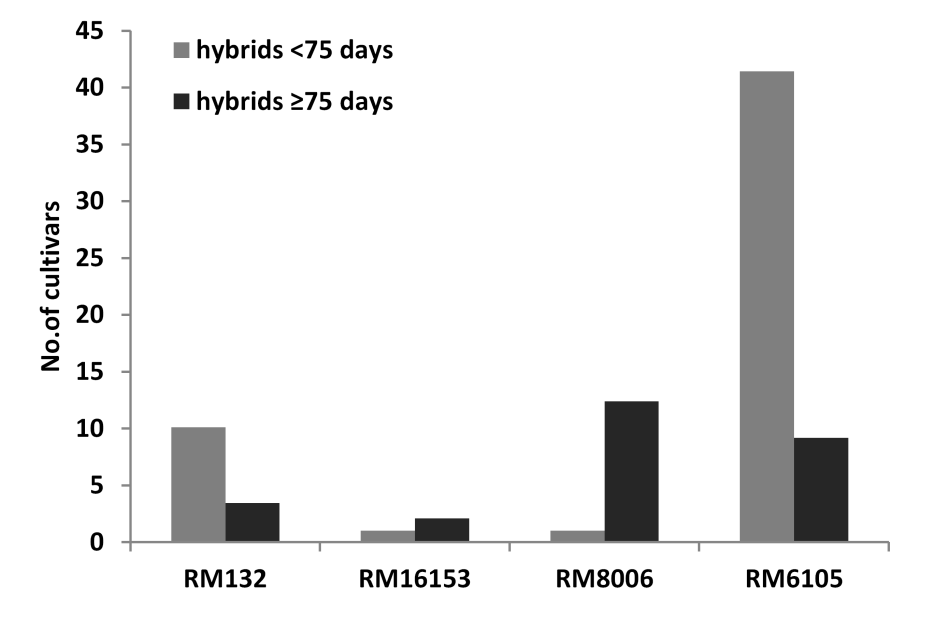
**

**Supplemental Figure 5. Distribution of four Cd-associated loci in the early maturating and late maturing groups**
